# Novel Nanoparticle-Based Treatment and Imaging Modalities

**DOI:** 10.3390/pharmaceutics15010244

**Published:** 2023-01-11

**Authors:** Oleh Taratula, Olena R. Taratula

**Affiliations:** Department of Pharmaceutical Sciences, College of Pharmacy, Oregon State University, Portland, OR 97201, USA

Over the last twenty years, nanomaterials have been widely used in cancer research. Numerous reports suggest that nanoparticles have the potential to improve conventional therapeutic (e.g., chemotherapy) and imaging (e.g., MRI) modalities for cancer detection and treatment [1]. Moreover, nanomaterials allow for the further development of novel experimental treatments and imaging strategies, including photothermal therapy, magnetic hyperthermia, and photoacoustic imaging [2,3,4,5]. In addition, although to a lesser extent, nanoparticle-based imaging and treatment modalities have been explored for other non-malignant diseases and disorders, such as ectopic pregnancy, endometriosis, muscle atrophy and several more [6,7,8]. It has been revealed that knowledge of cancer nanomedicine and some fundamental principles could potentially be used for the development of novel nanoparticle-based strategies for the treatment and imaging of other disorders [9]. In this Special Issue, researchers from eleven countries published four reviews and nine original research articles detailing recently developed nanoparticle-based modalities for the diagnosis and treatment of various disease states.

A significant number of published articles have been devoted to the application of novel nanomedicine platforms for various cancers, such as high-grade gliomas, Ewing’s sarcoma and others. For example, the report by Nazzaro et al., describes a novel drug molecule called ML111 that exhibits selectivity against Ewing’s sarcoma cells [10]. To overcome ML111’s limited aqueous solubility and improve its systemic delivery to cancer tumors, this drug candidate was encapsulated in the hydrophobic core of polyethylene glycol-poly(caprolactone) block co-polymer (PEG-PCL)-based nanoparticles. Following the intravenous injection of mice, the ML111-loaded PEG-PCL nanoparticles accumulated efficiently in Ewing’s sarcoma xenografts and significantly inhibited cancer growth. In another article, Formaggio et al., developed gold nanoparticles functionalized with two different therapeutic peptides (C7H2 and HuAL1) that exhibit antitumor activity [11]. In vivo studies on a murine model of metastatic melanoma revealed that gold nanoparticles significantly enhance the anticancer efficacy of C7H2 and HuAL1 by protecting these peptides from degradation and simultaneously delivering them to the tumor site. Aldawsari et al., also reported that gold nanoparticles stabilized with Gum acacia could be used as a carrier for hydrophobic Letrozole, a clinically approved inhibitor for the treatment of breast cancer in postmenopausal women [12]. In addition, Mahmoud et al., demonstrated that polycaprolactone nanoparticles have the potential to deliver the hydrophilic drug irinotecan hydrochloride trihydrate (IRH) to primary human high-grade glioma cells over a sustained period of time, and presented a novel method for improving IRH encapsulation efficiency within these nanoparticles [13].

Aside from their use as drug delivery vehicles, several reports have shown that nanoparticles themselves can serve as anticancer therapeutic and imaging agents. Albarqi et al., presented a novel nanoparticle treatment modality for prostate cancer based on systemically delivered magnetic hyperthermia [14]. The authors designed biocompatible clusters of zinc and manganese-doped iron oxide nanoparticles with enhanced heating efficiency, which significantly inhibit tumor growth by accumulating in prostate cancer xenografts following intravenous injection, and elevating the intratumoral temperature above 42 °C in the presence of an alternating magnetic field. Huang et al., further provide a comprehensive overview of the role of cobalt nanoparticles in the development of novel anticancer therapeutic strategies [15]. They discuss the therapeutic properties of these nanoparticles, including their ability to induce the selective inhibition of cancer cells, enhance the efficacy of immunotherapy, exhibit photothermal properties, sensitize cancer cells to chemotherapy, and reduce the side effects of chemotherapeutic drugs. Finally, Yu et al., reported novel multimodal nanoparticle-based imaging probes that simultaneously provide high temporal and spatial resolution to improve early cancer diagnosis [16]. They developed a simple hydrothermal method for the synthesis of ytterbium, gadolinium, and neodymium-doped calcium fluoride nanoparticles, capable of near infrared fluorescence, photoacoustic, and magnetic resonance imaging. 

This Special Issue includes a number of research and review articles highlighting the therapeutic and diagnostic potential of novel nanoparticle-based platforms for non-malignant disorders. In the first paper, Alonso-Alonso et al., reported the development of fibrin-targeted nanoparticles acting as T_1_ and T_2_ MRI contrast agents for the detection and diagnosis of small blood clots in acute ischemic stroke [17]. Animal studies revealed that these nanoparticles have an affinity for the fibrin content of blood clots, and that the combination of the T_2_ and T_1_ MRI signals improves the distinction between fresh and old blood clots. As fresh clots are more likely to be disrupted by recombinant tissue plasminogen activators and easily removed via mechanical thrombectomy, it was concluded that the developed nanoparticles could be used to predict the efficacy of recanalization treatment. Several articles in this Special Issue are also dedicated to the development and application of particle-based therapeutic platforms for neurodegenerative and neurological disorders. Kopach et al., described layer-by-layer (LbL)-fabricated microcapsules loaded with nerve growth factor (NGF) that guide the morphological development of hippocampal neurons in vitro [18]. It was suggested that the constructed LbL-microcapsules could be suitable for the delivery of NGF to specific populations of brain neurons. In another publication, Fay et al., presented a novel method for the preparation of PEG-free polyion complex nanocarriers for the delivery of brain-derived neurotrophic factor to its target receptor in the brain following intranasal administration [19]. When used for the treatment of neurodegenerative diseases, this new formulation is expected to have a lower immunogenic profile than the previously reported PEG-containing polyion complex. Crucially, this Special Issue is supplemented with a comprehensive review demonstrating that nose-to-brain delivery of therapeutic agents using nanoparticle-based carriers has great potential for the treatment of neurodegenerative diseases and other central nervous system disorders [20]. In this article, Lee et al., provided a detailed analysis of the current status of nose-to-brain delivery for nanotherapeutics from various perspectives, including mechanistic biology, transport kinetics, formulations, and clinical applications. 

This Special Issue concludes with two articles that offer a comprehensive overview of nanotoxicology and the characterization techniques used to control and predict the behavior of nanoparticle-based drug delivery systems. Alshawwa et al., discussed the benefits and limitations of commonly used methods for assessing various properties of nanocarriers, such as physicochemical parameters, stability, drug loading efficiency, tissue permeability, and so on [21]. Moreover, the authors discussed the current status and future prospects of the application of artificial intelligence to the development and optimization of nanocarriers. In another review article, Ahmad et al., summarized the trends and challenges in the assessment of nanocarrier safety and toxicity, both in vitro and in vivo [22]. A brief overview of the current clinical status of nanomedicine was also provided.

Overall, the articles published in this Special Issue demonstrate that nanotechnology can significantly improve clinically available therapeutic and imaging modalities and can serve as a foundation for the discovery of novel diagnostic and treatment strategies for a variety of diseases. The Guest Editors are sincerely grateful to all authors for their excellent research contributions, and to all the reviewers for their thorough evaluation of the submitted manuscripts. We also would like to express our gratitude to the Assistant Editor, Echo Ma, and to the Pharmaceutics team for their invaluable help.

## References

[1] Wicki A., Witzigmann D., Balasubramanian V., Huwyler J. (2015). Nanomedicine in Cancer Therapy: Challenges, Opportunities, and Clinical Applications. J. Control. Release.

[2] Schumann C., Chan S., Khalimonchuk O., Khal S., Moskal V., Shah V., Alani A.W., Taratula O., Taratula O. (2016). Mechanistic Nanotherapeutic Approach Based on siRNA-Mediated DJ-1 Protein Suppression for Platinum-Resistant Ovarian Cancer. Mol. Pharmaceut..

[3] Demessie A.A., Park Y., Singh P., Moses A.S., Korzun T., Sabei F.Y., Albarqi H.A., Campos L., Wyatt C.R., Farsad K. (2022). An Advanced Thermal Decomposition Method to Produce Magnetic Nanoparticles with Ultrahigh Heating Efficiency for Systemic Magnetic Hyperthermia. Small Methods.

[4] Duong T., Li X., Yang B., Schumann C., Albarqi H.A., Taratula O., Taratula O. (2017). Phototheranostic Nanoplatform Based on a Single Cyanine Dye for Image-Guided Combinatorial Phototherapy. Nanomedicine.

[5] Lorenz A.S., Moses A.S., Mamnoon B., Demessie A.A., Park Y., Singh P., Taratula O., Taratula O. A Photoacoustic Contrast Nanoagent with a Distinct Spectral Signature for Ovarian Cancer Management. *Adv. Healthc. Mater.*
**2022**, e2202946. https://onlinelibrary.wiley.com/doi/10.1002/smll.202202343.

[6] Schumann C., Nguyen D.X., Norgard M., Bortnyak Y., Korzun T., Chan S., Lorenz A.S., Moses A.S., Albarqi H.A., Wong L. (2018). Increasing Lean Muscle Mass in Mice via Nanoparticle-Mediated Hepatic Delivery of Follistatin mRNA. Theranostics.

[7] Moses A.S., Taratula O.R., Lee H., Luo F., Grenz T., Korzun T., Lorenz A.S., Sabei F.Y., Bracha S., Alani A.W.G. (2020). Nanoparticle-Based Platform for Activatable Fluorescence Imaging and Photothermal Ablation of Endometriosis. Small.

[8] Moses A.S., Kadam L., St Lorenz A., Baldwin M.K., Morgan T., Hebert J., Park Y., Lee H., Demessie A.A., Korzun T. Nano-Theranostic Modality for Visualization of the Placenta and Photo-Hyperthermia for Potential Management of Ectopic Pregnancy. *Small*
**2022**, e2202343. https://onlinelibrary.wiley.com/doi/abs/10.1002/smll.202202343.

[9] Moses A.S., Demessie A.A., Taratula O., Korzun T., Slayden O.D., Taratula O. (2021). Nanomedicines for Endometriosis: Lessons Learned from Cancer Research. Small.

[10] Esfandiari Nazzaro E., Sabei F.Y., Vogel W.K., Nazari M., Nicholson K.S., Gafken P.R., Taratula O., Taratula O., Davare M.A., Leid M. (2021). Discovery and Validation of a Compound to Target Ewing’s Sarcoma. Pharmaceutics.

[11] Formaggio D.M.D., Magalhaes J.A., Andrade V.M., Conceicao K., Anastacio J.M., Santiago G.S., Arruda D.C., Tada D.B. (2022). Co-Functionalization of Gold Nanoparticles with C7H2 and HuAL1 Peptides: Enhanced Antimicrobial and Antitumoral Activities. Pharmaceutics.

[12] Aldawsari H.M., Singh S., Alhakamy N.A., Bakhaidar R.B., Halwani A.A., Badr-Eldin S.M. (2021). Gum Acacia Functionalized Colloidal Gold Nanoparticles of Letrozole as Biocompatible Drug Delivery Carrier for Treatment of Breast Cancer. Pharmaceutics.

[13] Mahmoud B.S., McConville C. (2021). Development and Optimization of Irinotecan-Loaded PCL Nanoparticles and Their Cytotoxicity against Primary High-Grade Glioma Cells. Pharmaceutics.

[14] Albarqi H.A., Demessie A.A., Sabei F.Y., Moses A.S., Hansen M.N., Dhagat P., Taratula O.R., Taratula O. (2020). Systemically Delivered Magnetic Hyperthermia for Prostate Cancer Treatment. Pharmaceutics.

[15] Huang H., Wang J., Zhang J., Cai J., Pi J., Xu J.F. (2021). Inspirations of Cobalt Oxide Nanoparticle Based Anticancer Therapeutics. Pharmaceutics.

[16] Yu Z., He Y., Schomann T., Wu K., Hao Y., Suidgeest E., Zhang H., Eich C., Cruz L.J. (2022). Achieving Effective Multimodal Imaging with Rare-Earth Ion-Doped CaF(2) Nanoparticles. Pharmaceutics.

[17] Alonso-Alonso M.L., Perez-Mato M., Sampedro-Viana A., Correa-Paz C., Avila-Gomez P., Sobrino T., Campos F., Castillo J., Iglesias-Rey R., Hervella P. (2022). Fibrin-Targeted Nanoparticles for Finding, Visualizing and Characterizing Blood Clots in Acute Ischemic Stroke. Pharmaceutics.

[18] Kopach O., Pavlov A.M., Sindeeva O.A., Sukhorukov G.B., Rusakov D.A. (2020). Biodegradable Microcapsules Loaded with Nerve Growth Factor Enable Neurite Guidance and Synapse Formation. Pharmaceutics.

[19] Fay J.M., Lim C., Finkelstein A., Batrakova E.V., Kabanov A.V. (2022). PEG-Free Polyion Complex Nanocarriers for Brain-Derived Neurotrophic Factor. Pharmaceutics.

[20] Lee D., Minko T. (2021). Nanotherapeutics for Nose-to-Brain Drug Delivery: An Approach to Bypass the Blood Brain Barrier. Pharmaceutics.

[21] Alshawwa S.Z., Kassem A.A., Farid R.M., Mostafa S.K., Labib G.S. (2022). Nanocarrier Drug Delivery Systems: Characterization, Limitations, Future Perspectives and Implementation of Artificial Intelligence. Pharmaceutics.

[22] Ahmad A., Imran M., Sharma N. (2022). Precision Nanotoxicology in Drug Development: Current Trends and Challenges in Safety and Toxicity Implications of Customized Multifunctional Nanocarriers for Drug-Delivery Applications. Pharmaceutics.

